# Supplementary material for: Metabolic handoffs between multiple symbionts may benefit the deep-sea bathymodioline mussels
Source: ISME Commun. 2023 May 20;3:48. doi: 10.1038/s43705-023-00254-4 (PMC10199937; doi:10.1038/s43705-023-00254-4)
Supplement: Supplementary file 1 — Supplementary Information [file 43705_2023_254_MOESM1_ESM.pdf]

Supplementary Information

Metabolic handoffs between multiple symbionts may benefit the deep-sea bathymodioline mussels

Tal Zvi-Kedem[1,3], Simina Vintilla[2], Manuel Kleiner[2], Dan Tchernov[3], Maxim Rubin-Blum[1]

1: Morris Kahn Marine Research Station, Department of Marine Biology, Leon H. Charney School of Marine Sciences, University of Haifa, Haifa, 3498838 Israel

2: Department of Plant and Microbial Biology, North Carolina State University, Raleigh, NC, 27695, USA

3: Biology Department, National Institute of Oceanography, Israel Oceanographic and Limnological Research (IOLR), Haifa, 3108000 Israel

Correspondence: Maxim Rubin-Blum – mrubin@ocean.org.il

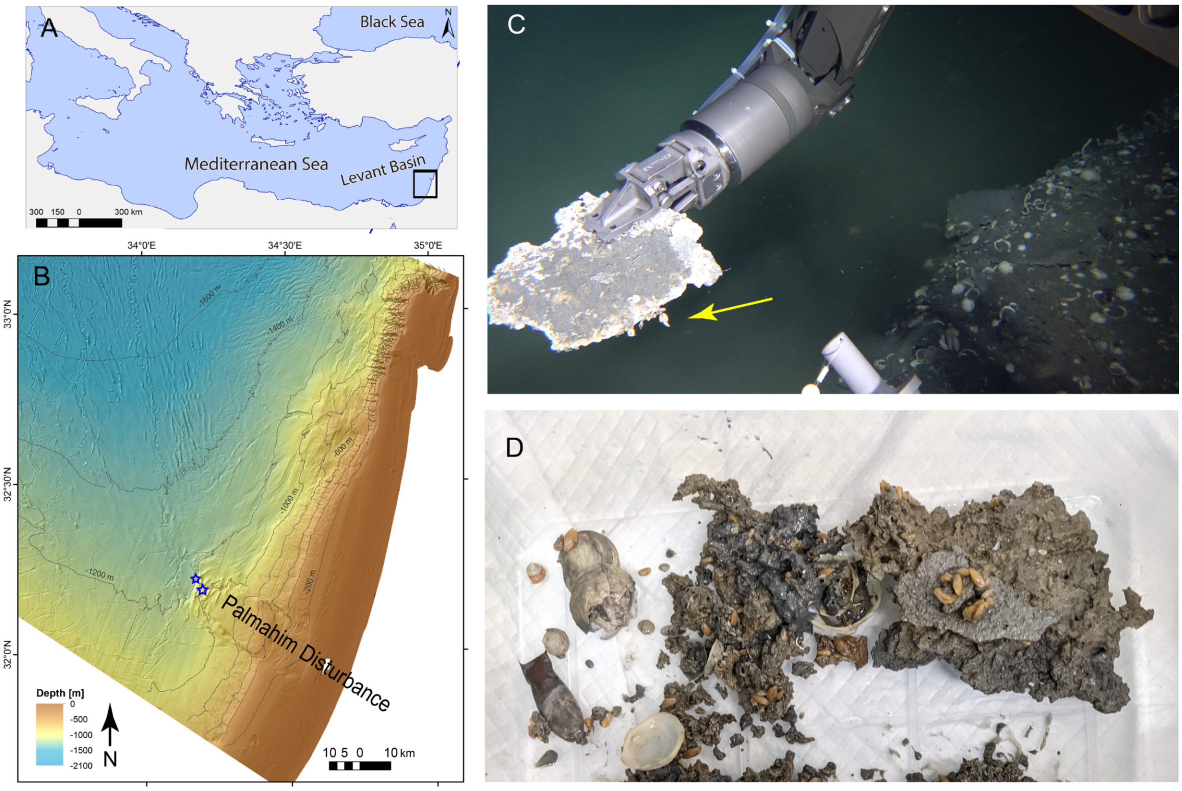

Figure S1: Collection sites of *Idas* specimens in the Eastern Mediterranean Palmahim Disturbance site (A,B, blue stars mark the two collection sites). *Idas* individuals were often found attached to authigenic carbonates (C- in situ, D – onboard).

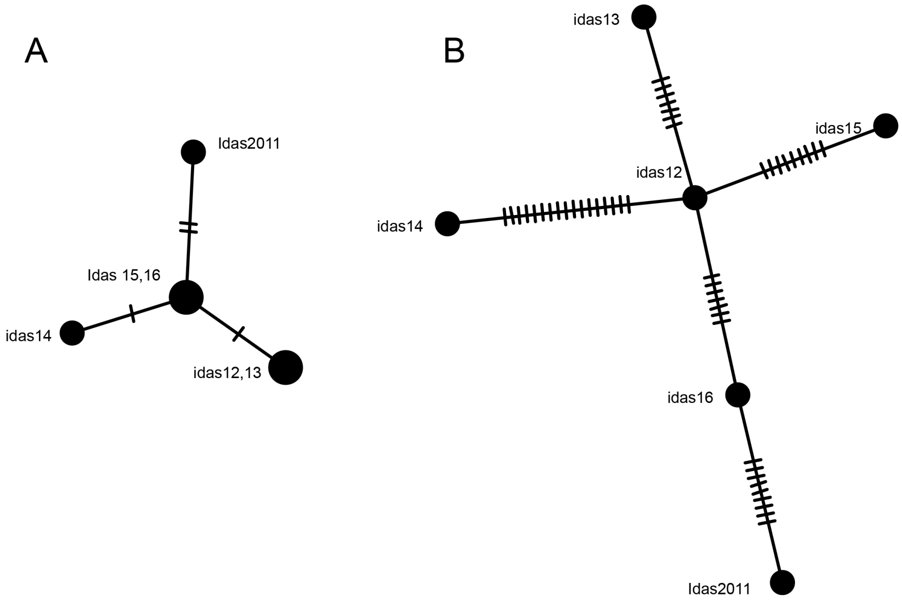

Figure S2: Mitochondrially encoded cytochrome c oxidase I (A) and mitochondrial genome (B) - based haplotype distances of the six *Idas modiolaeformis* individuals for which metagenomes were sequenced in this study. Each line indicates one single nucleotide polymorphism. The figure was produced with POPART (https://doi.org/10.1111/2041-210X.12410).

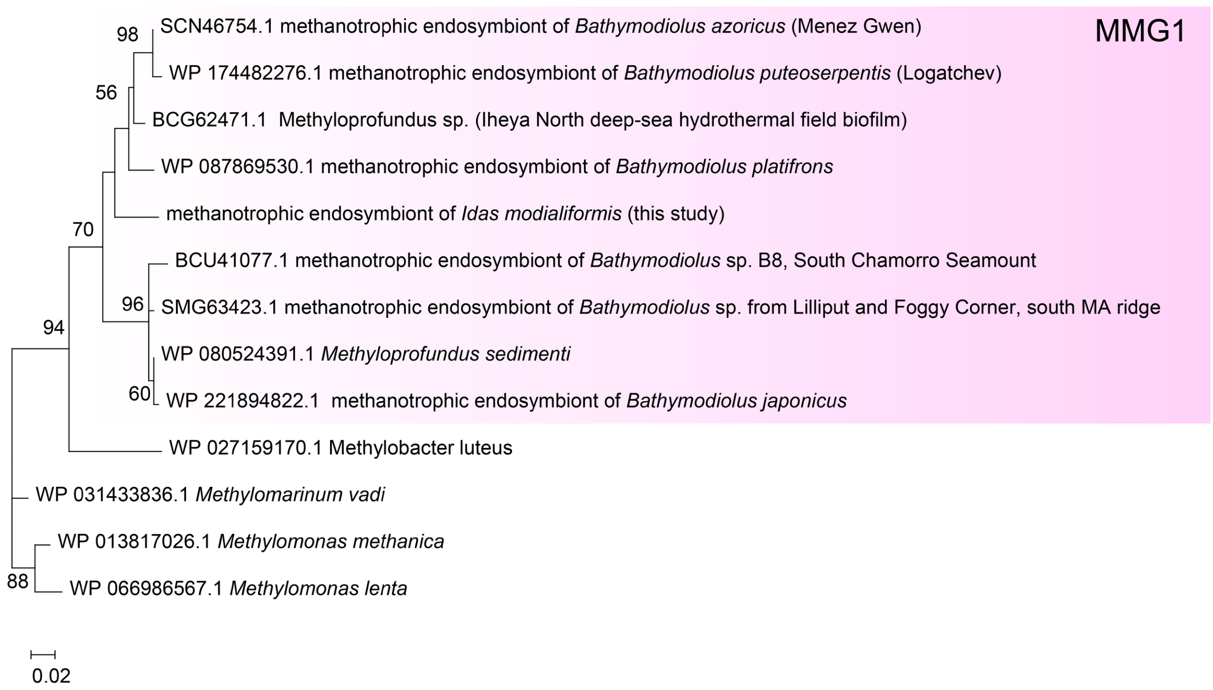

Figure S3: Phylogeny of the PmoA subunit of the particular methane monooxygenase of methane oxidizers including the methane-oxidizing symbiont of *Idas modiolaeformis*. The maximum likelihood midpoint-rooted tree is based on the LG model (MEGA11). The tree scale represents the number of substitutions per site.

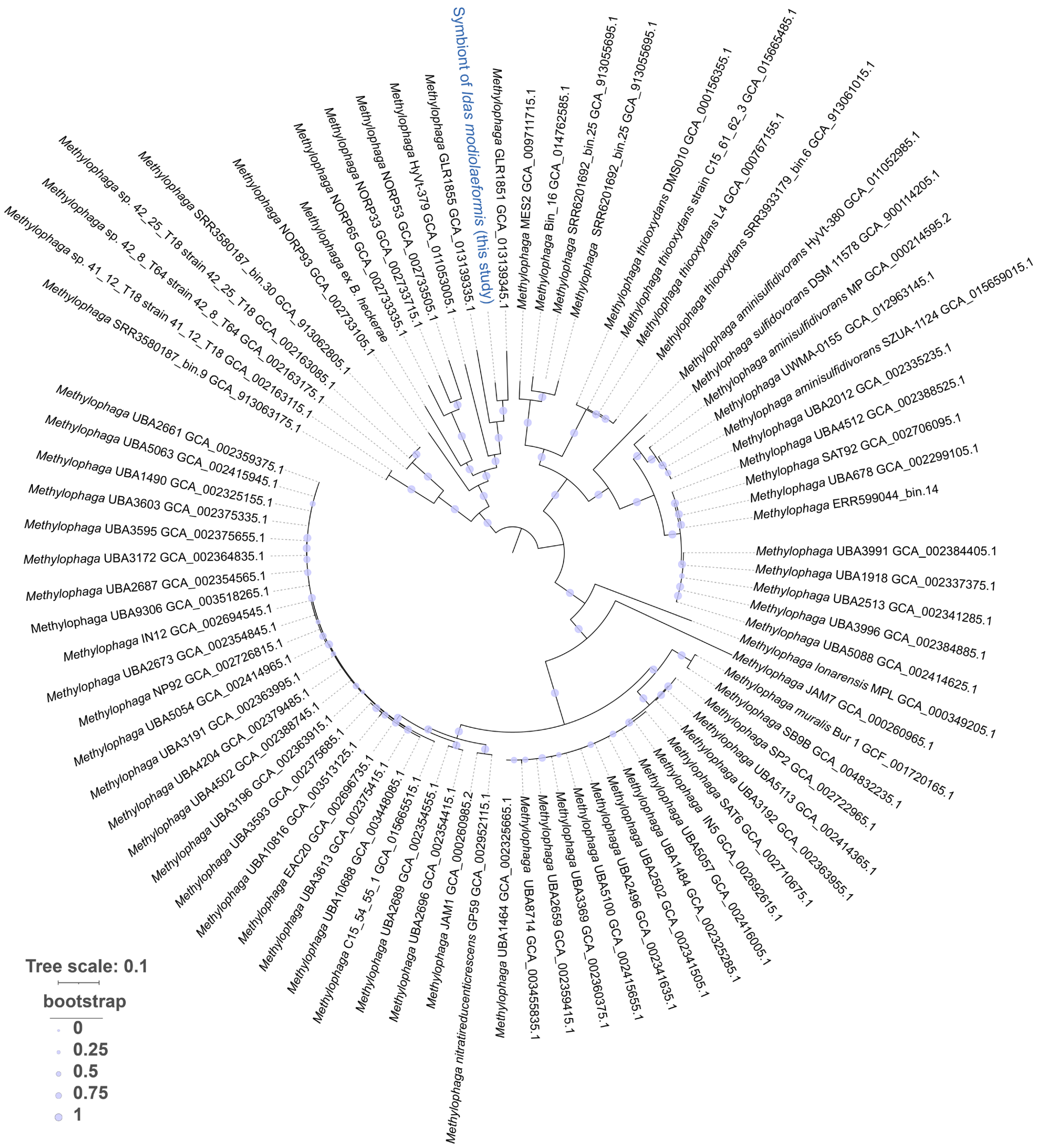

Figure S4: Phylogenomic tree of Methylophagaceae metagenome-assembled genomes, using the alignment of 172 protein sequences common to most gammaproteobacteria. The FastTree maximum likelihood tree was inferred using the JTT model, CAT approximation with 20 rate categories.

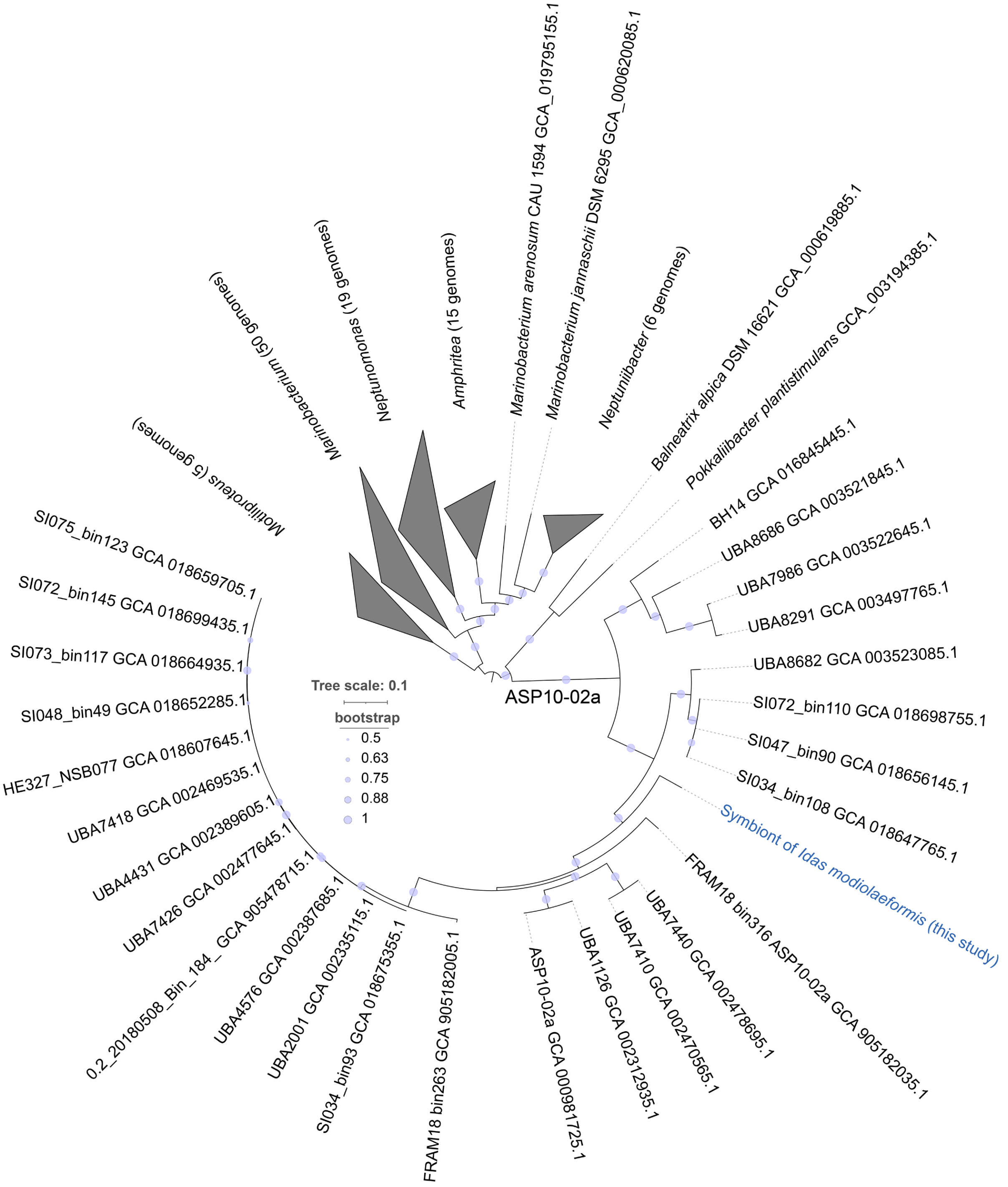

Figure S5: Phylogenomic tree of Nitrincolaceae metagenome-assembled genome, using the alignment of 172 protein sequences common to gammaproteobacteria. The FastTree maximum likelihood tree was inferred using the JTT model, CAT approximation with 20 rate categories.

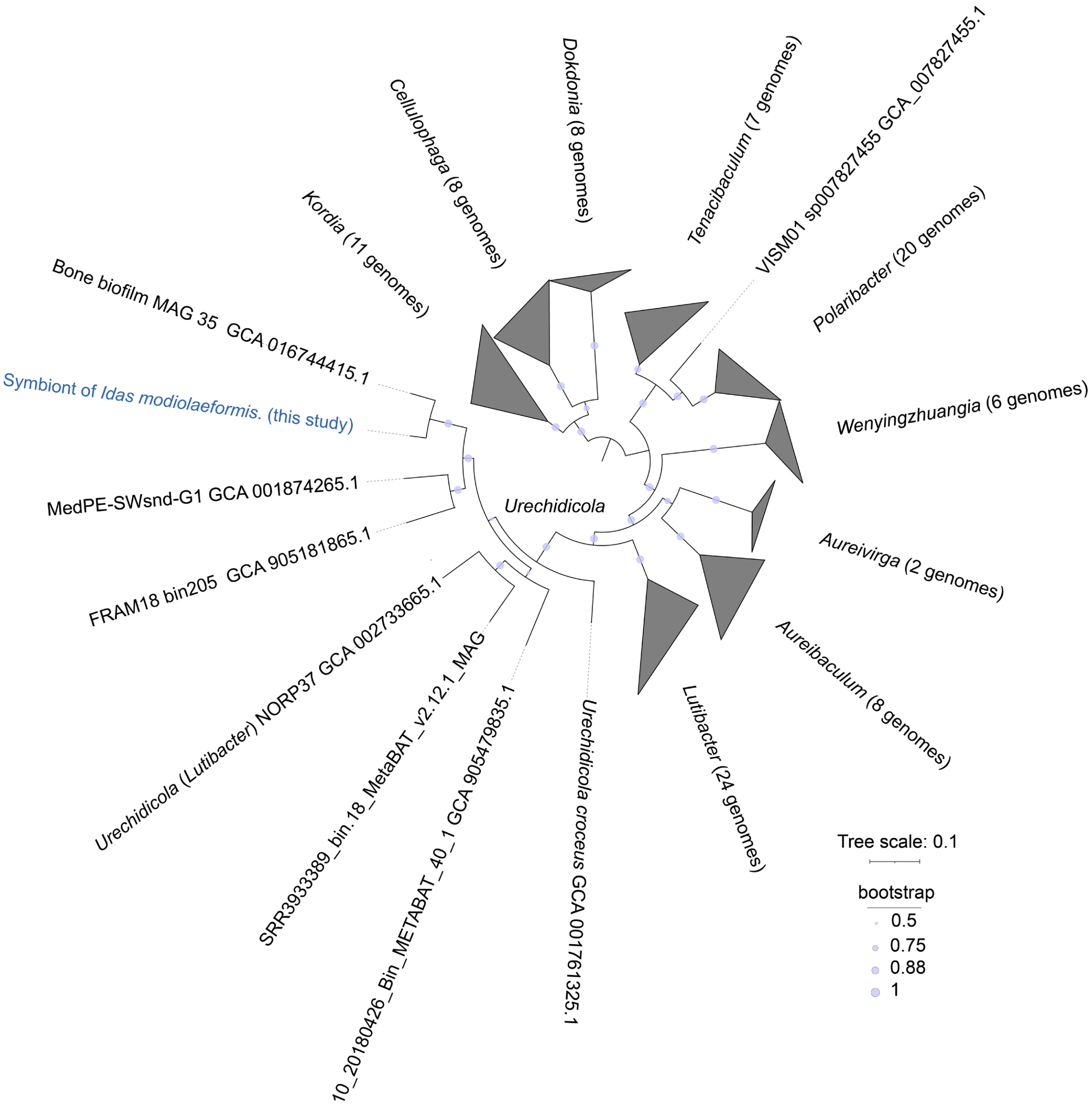

Figure S6: Phylogenomic tree of Flavobacteriaceae metagenome-assembled genome, using the alignment of 74 protein sequences common to bacteria. The FastTree maximum likelihood tree was inferred using the JTT model, CAT approximation with 20 rate categories.

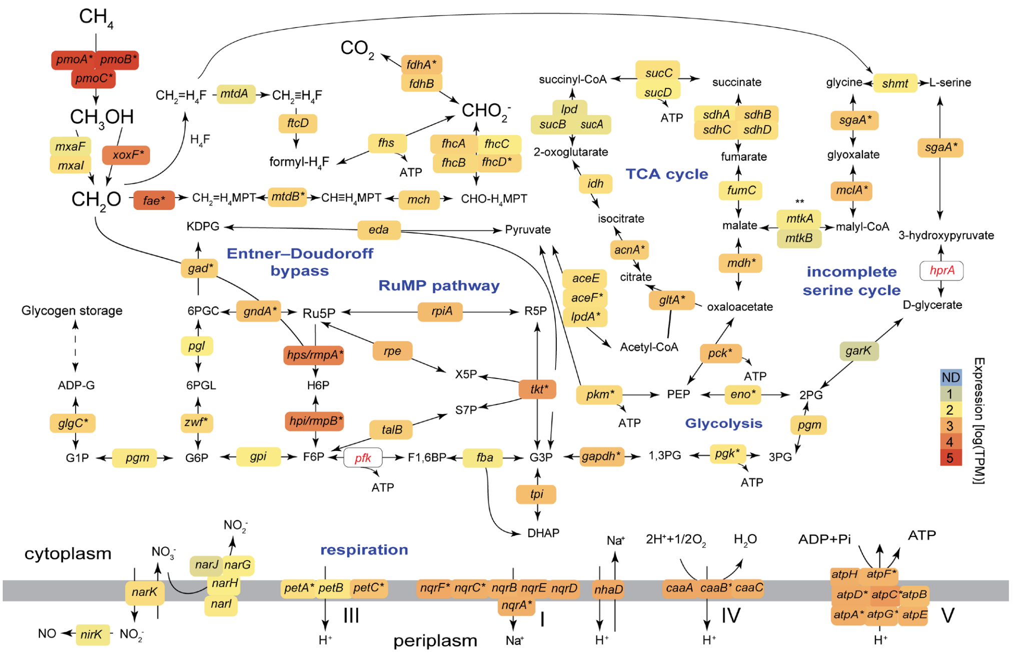

Figure S7: Central carbon metabolism in *Methyloprofundus* symbiont of *Idas modiolaeformis* and measured transcription levels in the metatranscriptomes. The *pfk* and *hprA* genes were not found in the MAG, therefore the Embden–Meyerhof–Parnas (EMP) variant of the ribulose monophosphate (RuMP) pathway is likely not present and the serine cycle is incomplete. The genes are as follows: calcium-dependant methanol dehydrogenase *mxaFI*; lanthandine-dependant methanol dehydrogenase *xoxF*; 3-hexulose-6-phosphate synthase *hps/rmpA*; 3-hexulose-6-phosphate isomerase *hpi/rmpB*; transketolase *tkt*; ribose-5-phosphate isomerase *rpiA*; phosphoribulokinase *prk*; ribulose-phosphate 3-epimerase *rpe*; transaldolase *talB*; ATP-dependent 6-phosphofructokinase *pfk*; fructose-1,6-bisphosphate aldolase/phosphatase *fbp*: glucose-6-phosphate isomerase *gpi*; glucose-6-phosphate 1-dehydrogenase *zwf*; 6-phosphogluconolactonase *pgl*; phosphogluconate dehydratase *edd*; 2-dehydro-3-deoxy-phosphogluconate/2-dehydro-3-deoxy-6-phosphogalactonate aldolase *eda*; phosphoglucomutase *pgm*; glucose-1-phosphate adenylyltransferase *glgC*; fuctose-bisphosphate aldolase *fba*; triosephosphate isomerase *tpi*; formaldehyde activating enzyme *fae*; methylene tetrahydromethanopterin dehydrogenase *mtdB*; methenyltetrahydromethanopterin cyclohydrolase *mch*; formyltransferase/hydrolase complex *fhcABCD*; NAD(P)-dependent methylenetetrahydromethanopterin dehydrogenase *mtdA*; bifunctional methylenetetrahydrofolate dehydrogenase / methenyltetrahydrofolate cyclohydrolase *fold*; formate--tetrahydrofolate ligase *fhs*; formate dehydrogenase *fdhAB*; glyceraldehyde-3-phosphate dehydrogenase *gapdh*; phosphoglycerate kinase *pgk*; phosphoglucomutase *pgm*; enolase *eno*; phosphoenolpyruvate synthase *ppsA*; phosphoenolpyruvate carboxykinase *pck*; pyruvate dehydrogenase *aceEF-lpdA*; pyruvate kinase *pkm*; citrate synthase *gltA*; aconitase *acnA*; isocitrate dehydrogenase *idh*; 2-oxoglutarate dehydrogenase complex *sucAB-lpd*; succinate--CoA ligase *sucCD*; succinate dehydrogenase *sdhABC*; fumarate hydratase class II *fumC*; malate hydrogenase *mdh*; malate--CoA ligase *mtkAB*; malyl-CoA lyase *mclA*; serine--glyoxylate aminotransferase *sgaA*; serine hydroxymethyltransferase *shmt*; glycerate dehydrogenase *hprA*; glycerate 2-kinase *garK*; caa_3-type cytochrome c oxidase *cyoABC*; ubiquinol-cytochrome c reductase *petABC*; Na(+)-translocating NADH-quinone reductase *nqrA-F*; Na(+)/H(+) antiporter *nhaD*; respiratory nitrate reductase *narGHIJ*, copper-containing nitrite reductase *nirK*; nitrate/nitrite antiporter *narK*; ATP synthase *atpA-F*. Metabolites: OA, oxaloacetate; PEP, phosphoenolpyruvate; 2-phosphoglycerate, 2PG; 3-phosphoglycerate, 3PG; 1,3-bisphosphoglycerate 1,3BPG; 3-phosphoglyceraldehyde, G3P; dihydroxyacetone phosphate, DHAP; fructose 1,6-bisphosphate, F1,6BP; fuctose 6-phosphate, F6P; hexulose 6-phosphate, H6P; ribulose 5-phosphate, Ru5P; ribulose-1,5-bisphosphate, Ru1,5BP; ribose 5-phosphate, R5P; glucose 6-phosphate, G6P; 6-phosphogluconolactonase, 6PGL; 2-Dehydro-3-deoxy-D-gluconate 6-phosphate, 6PGC; 2-keto-3-deoxy-6-phosphogluconate, KPDG; D-Xylulose 5-phosphate, X5P; sedoheptulose 7-phosphate, S7P; gucose 1-Phosphate, G1P; ADP-glucose, ADP-G; tetrahydrofolate, H_4F; tetrahydromethanopterin, H_4MPT. Average expression values from 4 individuals are shown.

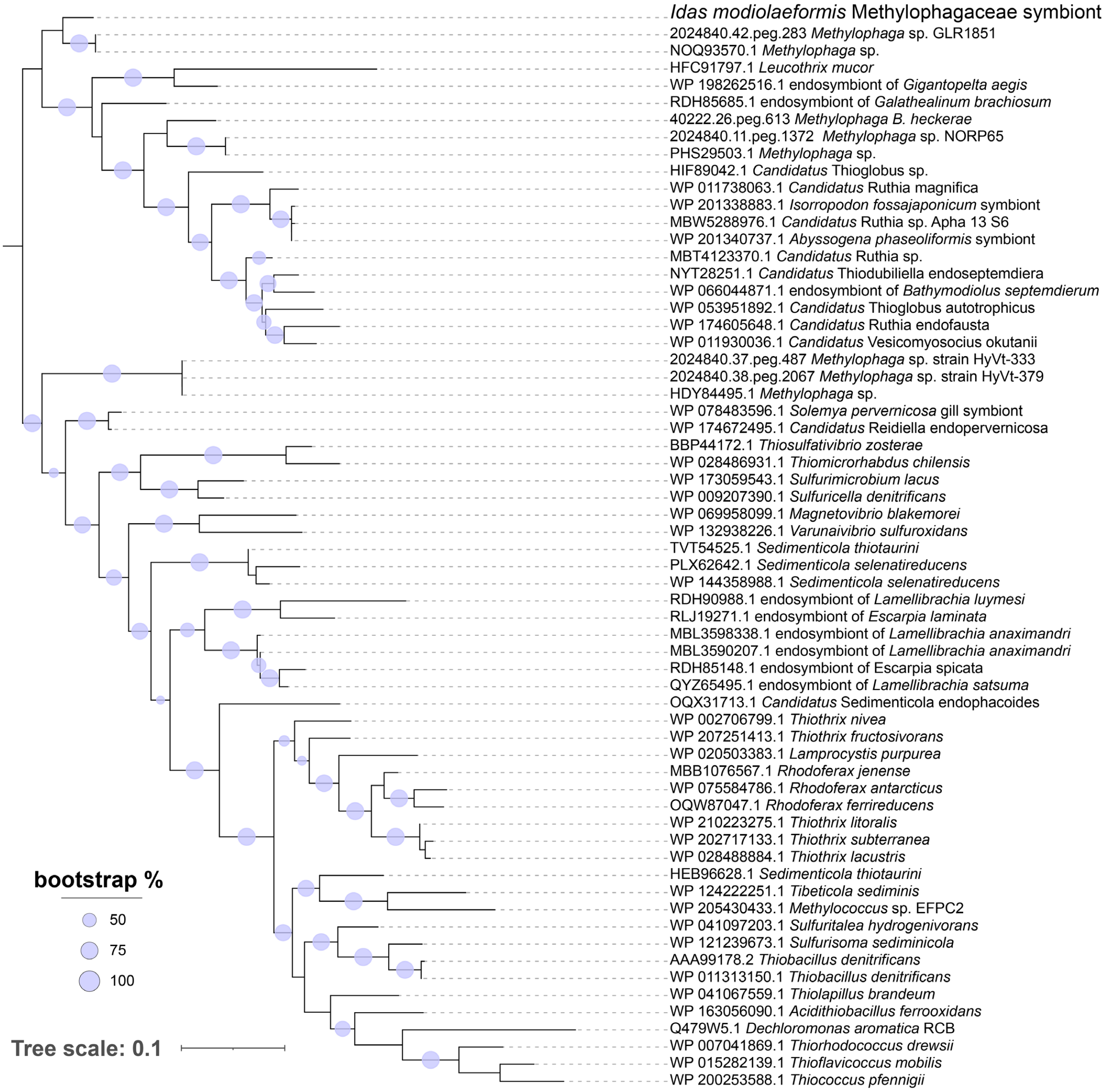

Figure S8: Phylogenetic tree of the CbbM (form II Ribulose-1,5-bisphosphate carboxylase-oxygenase, RuBisCO) amino acid sequences from symbiotic Methylophagaceae and selected bacteria. The maximum likelihood tree is based on the LG+I+G4 model (IQ-TREE 2). The tree scale represents the number of substitutions per site.

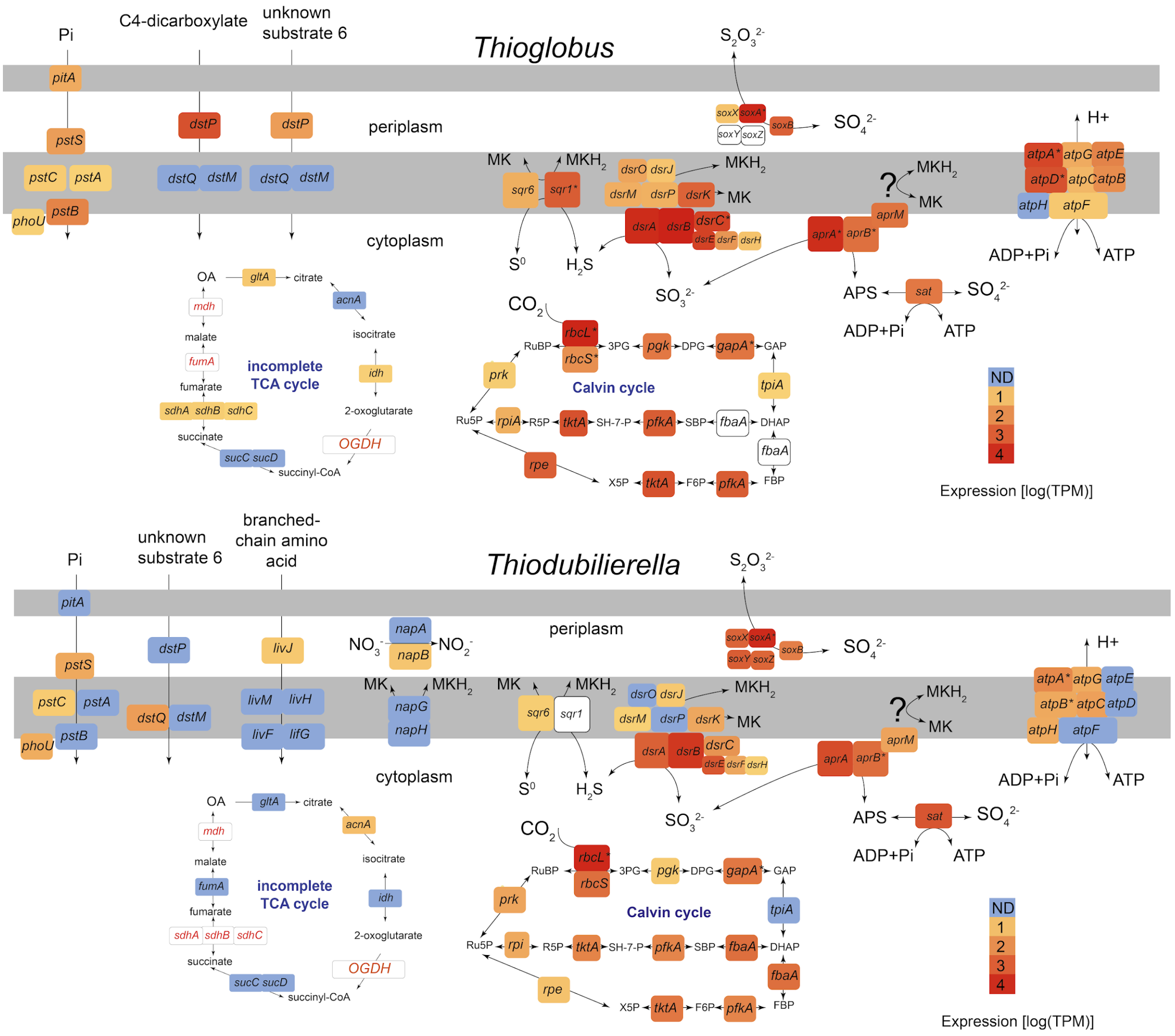

Figure S9: Central metabolism in the sulfur-oxidizing symbionts of *Idas modiolaeformis* (top panel: *Thioglobus*, bottom panel: *Thiodubilierella*). White boxes indicate functions that were not found. Expression levels shown correspond to transcription levels from the metatranscriptomic data. Average expression values from 4 individuals are shown. Proteins/genes: sulfur oxidation complex *soxAXYZB*; sulfide:quinone oxidoreductase, type I/VI, *sqr1* / 6; dissimilatory sulfite reductase *dsrAB*; sulfite reduction-associated complex *dsrMKJOP-C-EFH*; adenylylsulfate reductase subunit *aprABM*; ATP synthase *atpA-F*; ribulose bisphosphate carboxylase form I *rbcLS*; phosphoglycerate kinase *pgk*; glyceraldehyde-3-phosphate dehydrogenase *gapA*; triosephosphate isomerase *tpiA*; ribose-5-phosphate isomerase *rpi*; transketolase *tktA*; pyrophosphate-dependent fructose 6-phosphate-1-kinase *pfkA*; ribulose-phosphate 3-epimerase *rpe*; fructose-bisphosphate aldolase class *fbaA*; citrate synthase *gltA*; aconitase *acnA*; isocitrate dehydrogenase *idh*; 2-oxoglutarate dehydrogenase complex *OGDH*; succinate--CoA ligase *sucCD*; succinate dehydrogenase *sdhABC*; fumarate hydratase class I *fumA*; malate hydrogenase *mdh*. Metabolites: OA, oxaloacetate; 3-Phosphoglyceric acid, 3PG, glyceraldehyde 3-phosphate, GAP; 1,3-bisphosphoglycerate, DPG; dihydroxyacetone phosphate, DHAP; fructose 1,6-bisphosphate, FBP; fructose 6-phosphate, F6P; D-Xylulose 5-phosphate, X5P; sedoheptulose-1,7-bisphosphate, SBP; sedoheptulose 7-phosphate, SH-7-P; ribose 5-phosphate, R5P; Ru5P; ribulose-1,5-bisphosphate, RuBP. MK/MKH₂ represents the oxidized and reduced quinone pool. Pi is pyrophosphate.

Table S1 (Excel sheets): Genomic features of *Idas modiolaeformis* symbionts, and their RNA- and protein-level expression (raw mapped read counts and log[transcripts per million x 10^6] for RNA, % normalized spectral abundance factor, %NSAF for proteins). Each one of the six metagenome-assembled genomes is summarized in a separate tab. Coding sequence, as well as their protein-level translations, are included. Rast SEED annotations are shown.

Table S2 (Excel sheets): dbCAN annotation and RNA-level expression values of Carbohydrate-Active Enzymes (CAZymes) in the genomes of *Urechidicola* symbionts of *Idas modiolaeformis*.
